# The Association between Dairy Intake, Simple Sugars and Body ‎Mass Index with Expression and Extent of Anger in Female ‎Students ‎

**Published:** 2016-01

**Authors:** Naser Kalantari, Saeid Doaei, Maedeh Gordali, Ghazal Rahimzadeh, Maryam Gholamalizadeh

**Affiliations:** 1 Department of Nutrition and Food Sciences, Shahid ‎Beheshti University of Medical Sciences, Tehran, Iran; 2 Student’s Research Committee, National Nutrition and Food Technology Research Institute, Faculty of Nutrition Sciences and Food Technology, Shahid Beheshti University of Medical Sciences, Tehran, Iran.; 3 Department of Community Nutrition, Shahid Beheshti University of Medical Sciences, Tehran, Iran‎; 4 Department of Food Science and Technology, Science and Research Branch, Azad University, Tehran, ‎Iran; 5 Food Security Research Center, Department of Clinical Nutrition, School of Nutrition and Food Sciences, ‎Isfahan University of Medical Sciences, Isfahan, Iran

**Keywords:** Anger, Body Mass Index, Food Intake

## Abstract

**Objective: **A significant increase in violence in the world and its impact on public health and ‎society can be an important reason to offer solutions to reduce or control anger. Studies ‎have shown that specific food groups may be effective in controlling mental disorders ‎such as depression, anxiety and anger. The purpose of this study was to determine the ‎relationship between food intake and Body Mass Index on state-trait anger expression ‎in female students of Shahid Beheshti University of Medical Sciences. ‎

**Method:** In this cross-sectional study, 114 female students were randomly selected from ‎dormitories of Shahid Beheshti University of Medical Sciences. Body height and ‎weight were measured using the scale and stadiometer, respectively. The required data ‎for evaluating the relationship between state-trait anger expression and food ‎consumption groups were collected using State-Trait Anger Expression Inventory-2 ‎‎ (STAXI-2) and Food Frequency questionnaires.‎

**Results:** The results revealed a significant negative correlation between consumption of dairy ‎product and trait anger (angry reaction), (P = 0.015). This association remained ‎significant after adjustment of confounding factors. No significant correlations were ‎found between other food groups as well as BMI and state-trait anger expression.‎

**Conclusion**: The higher intake of dairy products reduced state-trait anger expression. This result is ‎consistent with the findings of many studies on the effect of dairy consumption on ‎mental disorders. Therefore, consumption of dairy products can be a solution for ‎reducing anger.‎

Anger has been characterized as a complex construct including hostility, irritability and ‎aggressive behavior (1). Anger can be defined as an emotional response or internal ‎feeling which is caused by physiological excitation, cognition and malice thoughts (2). ‎Nevertheless, anger is an emotion of satisfaction that prepares us to face the potential ‎surrounding risks. On the other hand, destructive impact of anger may have detrimental ‎effects on humans and their environments (3). Anger is a robust risk factor for Coronary ‎Heart Disease (CHD) (4, 5), Cancer (6), and Eating disorders (7) which can affect the ‎quality of life (8, 9). Demark et al. (2011) have reported that over 36% of patients with ‎brain injury have shown anger and aggression states (10). The effects of diet have been ‎reported on depression (11), fervour (12), brain function (13, 14), stress response system ‎‎(15), and oxidative reactions (16). On the other hand, many people eat to overcome ‎emotional problems, mental pressure and anxiety (17, 18). There has been a positive ‎correlation between obesity and some mental disorders including depression and anxiety (19, 20). Eating disorders in response to mental stress, fatigue, anxiety, stress and ‎depression have been reported (21). ‎

Many studies found that lack of micronutrients such as Zinc (22), vitamin E, Iron, ‎Calcium, Iodine, Selenium, Folic acid (23), vitamin C (24), Choline (25), and vitamin D (26) is common among students all over the world. On the other hand, residency in ‎dormitories can exacerbate the nutritional risk factors among students. Previous study ‎showed that nutritional status of students living in the dorms were different from other ‎students. Food consumption in dormitory students was decreased when entering ‎university (27) and significant drop in food intake quality was observed in half of the ‎dormitory students (28). Furthermore, despite the lack of some micronutrients intake, ‎the nutritional status of students who were not living in the dorms were more acceptable ‎compared to the dormitory students (29).‎

Previous studies about the effects of food and nutrient intake on mental disorders ‎illustrated that a high intake of low-fat dairy products (30), decrease in consumption of ‎fast food (31), using the Mediterranean Diet (32), and consumption of omega-3 fatty ‎acids (33) and magnesium (34) supplements can result in reducing depression, anxiety ‎and stress.‎

Further studies that investigated the relationship between food consumption and mental ‎conditions have considered the prevalence of depression and its relation to food intake ‎‎ (35, 36). Although anxiety has been a common problem among students (37), no study ‎was conducted on the relationship between diet and stressful situations (such as anger). ‎Considering the results of studies conducted on the relationship between dietary intakes ‎and mental disorders, we chose two food groups for evaluation.‎

Therefore, the purpose of this research was to determine the relation between dietary ‎intake of dairy foods, simple sugars and BMI with states of anger in female students of ‎a dormitory in Shahid Beheshti University of Medical Sciences, Tehran, Iran.‎

## Materials and Method

In this cross-sectional study, 114 resident students accommodating Fatima Zahra ‎dormitory at Shahid Beheshti University of Medical Sciences were randomly selected ‎and examined. This study was conducted from spring 2014 to autumn 2015.‎

Initially, students' weight and height were measured using Seca Scales (precision of 1 ‎kg) and a tape measure (accuracy of 1 cm), respectively. After calculating the Body ‎Mass Index, each participant was asked to complete the Anger Questionnaire, designed ‎by Spielberger, validated by Barabadi in Iran, to measure anger in teens and adults (38). ‎Spielberger and Brenner introduced this scale in 2003 and published it in 2009 to ‎evaluate experience, expression, control and taming of anger (39). This questionnaire consists ‎of 35 items, 4 subscales and 3 sections; the first part has 10 items, the second part ‎contains 10 items, and the third part has 15 items, which evaluate anger state, anger trait ‎and expression and control of anger, respectively. All sections of the questionnaire were ‎calibrated using 3-point Likert scale. ‎

To score the first section, the statements were identified as "never, a little, and very ‎high" and scored as 1, 2, 3, respectively. To score the second section and 5 questions of ‎the third section (which represents the expression of anger), the statements were ‎identified as "very little, sometimes, and often" and scored as 1, 2, 3, respectively. To ‎score the remaining questions in the third section (which represent the control of anger), ‎scoring was done inversely, namely the score of "very little" was 3, the statement of ‎‎"sometimes" was scored as 2, and the phrase "often" was awarded a score of 1 (40). The ‎first part with the phrase "I feel right now ..." measured the state of anger (41) which ‎graded the intensity of feeling. This section with 10 items included the anger state and ‎subscales cases as follows: (a) Sense of Furiously (42), (b) Willingness to Verbal ‎Expression of Anger (43), and (c) Trend to Physical Expressions of Anger (44). The ‎second section, entitled "I feel usually…. "Involved 10 items to measure trait anger (45), ‎and was graded as the first part. The scale of trait anger has two subscales of angry state ‎‎ (46) and angry reaction (47). The title of the third section was "What is your behavioral ‎reactions in angry mood?" that measured the expression and control of anger, and ‎included four scales that involved subscales as follows: (a) Outer anger impart (48), (b) ‎inner’s anger impart (49), c: Control of outer anger (50) and (d) control of inner anger ‎‎(51). ‎

The food frequency questionnaire was used to investigate the intake of dairy food ‎groups and simple sugars. This questionnaire was used to assess the frequency of food ‎intake in a day, week or month. To facilitate the evaluation of this method, foods were ‎organized as groups with similar nutrients.‎‏ ‏Since Food Frequency Questionnaire is based ‎on frequency of food intake (not only specific nutrient), the obtained information was ‎related to specific food group not specific nutrients (52). All participants signed the ‎informed consent to participate in the study.‎

Linear regression was used for data analysis. Data were recorded as mean±standard ‎deviation (SD). A statistical package (SPSS, version 21.0 for Windows, SPSS Inc.) was ‎used for data processing. Differences were considered significant at p <0.05.‎‎

## Results

The personal characteristics of the study population are shown in Table 1. In the current ‎study, 70% of the population aged between 20 to 25 years old. With respect to the ‎academic degree, they were divided into three groups of Bachelor, MSc, and PhD. The ‎number of students studying in each of these three groups was equal. In this community, ‎the BMI was 21.61±2.79 that was in the normal range. 13% of the Body Mass Index ‎was less than 18.5; and 75% of the Body Mass Index was between18.5 to 24.9, and ‎‎12% of the body mass index was greater than or equal to 25, which was considered as ‎overweight or obese. ‎

With regards to anger measurements, for the first section, the mean of calculating the ‎score was 13 out of 30. For trait anger, the calculated score was a little more than anger ‎state (18.75 of 30). On the other hand, the resulting score for the third section (anger ‎expression) was relatively high (28.35 of 45). ‎

With respect to food groups intake, the average consumption of dairy products in this ‎study was approximately 4 servings per day. According to the values recommended for ‎adults, intake of dairy products should be at least 2 servings per day (52). Therefore, the ‎dairy intake of the study population was averagely more than the minimum ‎recommended values. The mean intake of simple sugars was approximately 7 servings ‎per day that was higher than allowable values (0-3 servings) (52).‎

In this study, a significant inverse relationship was found between dairy consumption ‎and trait anger (reacted furiously and angry state) (P = 0.015). On the other hand, there ‎was not a significant correlation between dairy intake and simple sugars with other ‎subscales of anger (state and expression of anger). Furthermore, there was a negative ‎relevance between age and anger trait by investigating each parameters of age, height, ‎weight, and Body Mass Index and its relationship with anger (P=0.028). Overall, age ‎increase from 20 to 36 reduced levels of trait anger. ‎

No significant relevance was found between Body Mass Index and other subscales of ‎anger (state and expression of Anger). The relevance between dairy consumption, simple ‎sugars, age and anthropometric indices with all anger subscales is demonstrated in Table ‎‎2.‎

One of the confounding variables in this study was supplements intake by students. To ‎eliminate the effect of confounding variables and measuring the effect of calcium ‎supplementation on the relationship between anger and dairy intake, all persons who ‎were consuming calcium supplements or multivitamin-mineral (14) were excluded from ‎the study, and the results were reviewed. After the removal of the confounding ‎variables, the relationship between dairy consumption and trait anger remained ‎significant (P = 0.027). Table 3 displays the relationship between dairy consumption and ‎trait anger after removing the confounding variables.‎

## Discussion

There was a negative correlation between dairy intake (on levels recommended by the ‎Food Guide Pyramid) and trait anger (reacted furiously and angry state) (P-value = ‎‎0.015). Therefore, students who had more dairy products were less in angry states and ‎had low angry reactions. Miyake et al. reported similar results and showed that more ‎consumption of yogurt and calcium was associated with less rampancy of depression ‎symptoms (53). Crichton et al. reported that consumption of some low-fat dairy ‎products had beneficial effects on social functioning, stress and memory (54). Moreover, ‎Roohafza et al. found that more consumption of dairy products respond to low stress ‎levels (55). This effect may be due to the high content of tryptophan (essential amino ‎acid) in dairy products. Tryptophan is a precursor for serotonin (a neurotransmitter in ‎brain) that has a significant effect on mental states. In most previous studies, levels of ‎brain’s serotonin in patients with depression and anxiety were lower than healthy ‎subjects (56). Duke (57) and Giessen et al. (58) showed that serotonin function has an ‎inverse correlation with the degree of anger and hostility. Moreover, this effect can be related to the antioxidant content of dairy products such as ‎vitamins A and B2^1^ (57).

Antioxidants can affect oxidative stress, which may lead to ‎slower neuronal changes that are associated with depression in old age (59).‎

In this research, no significant correlation was found between intake of simple sugars, ‎and the expression and extend anger subscales; these results were also confirmed in ‎other studies. On the other hand, Keith et al. found that low-carbohydrate diet in female ‎group is associated with depression and anger (60). In the current study, consumption of ‎simple sugars (not total carbohydrate) in the long-term (1 year) was evaluated, and these ‎factors may cause the differences in the results. There was not a significant effect of ‎Body Mass Index on expression and extent of observed anger. ‎

Similarly, Arian et al. (61) and Ahmadi et al. (62) investigated that the prevalence of ‎mental health problems had no significant relationship with anthropometric indices ‎‎ (including BMI).‎

No association was found between BMI and depression in any other similar studies (63-‎‎^65^). However, in some cases, a significant positive correlation was detected between ‎depression and Body Mass Index (66-69). These differences in the results can be related ‎to several factors such as age, gender, socioeconomic and cultural factors that affect this ‎relationship. Furthermore, social attitudes to obesity should be considered as a ‎important cultural factor (70).For example, in Western culture, negative attitudes toward ‎obesity can influence self-esteem and cause depression (71). While in some other ‎societies larger body size is accepted (72).‎

^1^Vitamin B2 is one of many nutrients required to recycle glutathione, which is ‎one of the most important antioxidants in the human body. From a chemical ‎standpoint, what B2 does is facilitate the conversion of oxidized glutathione ‎into reduced glutathione. 

## Limitations

Since only female students and limited dormitories were evaluated in this study, ‎conducting similar research with larger samples is required. In addition, investigation of ‎other food groups associated with anger is a suggestion that can be offered in relation to ‎this research. The findings of this study are based on cross-sectional data, and a ‎important avenue for future research will be to replicate these studies with causal ‎structures using longitudinal data.‎

## Conclusion

Life quality can be affected by mental health as well as physical health. According to ‎the results of this study, more consumption of dairy food group decreases the trait ‎anger. Since the subscale of trait anger included angry state and angry reaction, it can be ‎concluded that dairy products intake has a positive effect on both temperament during ‎long time and immediate reaction to oppositions. Therefore, intake of dairy products can ‎be suggested as a nutritional strategy for reducing anger and increasing soothe. ‎

**Table 1:** The Basic Characteristics of the Participants

**Index**	**Mean**	**Standard deviation**
High (m)	1.63	0.047
Weigh (kg)	58.12	7.95
Body mass index (kg/m^2^)	21.61	2.79
Anger state (score of 30)	12.98	3.53
Trait Anger (score of 30)	18.75	4.01
Anger expression (score of 45)	28.37	6.36
Dairy consumption (serving per day)	3.89	2.06
Intake sugar (serving per day)	7.36	6.52

**Table 2:** Relationship between Dairy Consumption and Simple Sugars, Age and Anthropometric Indices with Anger

	**Anger State**			**Trait anger**			**Anger expression**		
**Index**	**B Coefficient**	**Beta coefficient**	**P-value**	**B Coefficient**	**Beta coefficient**	**P-value**	**B Coefficient**	**Beta coefficient**	**P-value**
**Dairy intake**	0.000	0.032	0.753	-0.001	-0.23	0.015*	-0.001	-0.105	0.273
**Sugar intake**	-6.42	-0.043	0.651	0.000	0.062	0.5	-8.80	-0.033	0.729
**Age**	-0.84	-0.074	0.445	-0.271	-0.210	0.028*	0.091	0.044	0.640
**Body mass index**	1.636	1.292	0.472	-0.391	-0.272	0.877	-7.021	-3.075	0.083
**High**	42.23	0.561	0.485	-12.05	-0.141	0.858	-190.004	-1.401	0.079
**Weight**	-0.594	-1.333	0.481	0.176	0.348	0.851	2.521	3.140	0.094

**Table 3:** The Relationship between Dairy Consumption with Trait Anger after Excluding 14 Patients

**Index**	**B Coefficient**	**Beta coefficient**	**P-value**
Dairy intake	-0.001	-0.221	0.027*
